# Hyaluronic Acid and Radiofrequency in Patients with Urogenital Atrophy and Vaginal Laxity

**DOI:** 10.3390/ph15121571

**Published:** 2022-12-16

**Authors:** Piotr Kolczewski, Mirosław Parafiniuk, Piotr Zawodny, Rashad Haddad, Agnieszka Nalewczyńska, Agnieszka Kinga Kolasa, Barbara Wiszniewska, Sophie Menkes, Alexander Bader, Giorgio Stabile, Nicola Zerbinati

**Affiliations:** 1 Department of Reconstructive Surgery and Gynecological Oncology, Pomeranian Medical University in Szczecin, Al. Powstańców Wielkopolskich 72, 70-111 Szczecin, Poland; 2 Szczecin Forensic Medicine Department, Pomeranian Medical University, 70-204 Szczecin, Poland; 3 Private Clinic “Zawodny Clinic” Szczecin, 71-047 Szczecin, Poland; 4 Obstetric Gynecology Faculty of the American Academy Aesthetic Gynecology Dubai-Paris-Riyadh, 491C Jumeirah Road, Dubai P.O. Box 566297, United Arab Emirates; 5 Private Clinic “Estebelle”, 02-972 Warszawa, Poland; 6 Histology and Embryology Department, Pomeranian Medical University, 70-204 Szczecin, Poland; 7 Clinique Nescens, 1272 Genolier, Switzerland; 8 Bader Medical Institute of London, 75 Harley Street, London W1G 8QL, UK; 9 Dermatology Clinic, Departmente of Medicine and Surgery, Università Vita-Salute San Raffaele, 20132 Milan, Italy; 10 Department of Medicine and Surgery, University of Insubria, 21100 Varese, Italy

**Keywords:** hyaluronic acid, radiofrequency, urogenital atrophy, vaginal atrophy, vaginal laxity

## Abstract

Vaginal laxity (VL) and genitourinary syndrome of menopause (GSM), as well as aesthetic changes in the vulvar skin, often occur together and cause physical, psychological, and functional problems for women and their partners. The current study evaluated the efficacy of a nonsurgical radiofrequency device (RF) procedure combined with hyaluronic acid (HA) injection into the skin of the labia majora on clinical, histological, and aesthetic levels. Twenty women with GSM and VL, aged between 36 and 72 (mean age 53.4), were treated with bipolar RF SECTUM, vaginal and vulvar application, as well as with a hyaluronic acid (HA) injection into the skin of the labia majora. The Vaginal Laxity Questionnaire (VLQ), Vaginal Health Index (VHI), and Female Sexual Function Index (FSFI) were used to examine the clinical effects of the operations. The Global Aesthetic Improvement Scale was utilized to measure patient satisfaction. On a histochemical level, the concentrations of elastin and collagen in the vaginal wall and vulvar skin were examined. Results: There was significantly higher patient satisfaction and a considerable clinical improvement across all areas of analysis. On the histochemical level, elastin and collagen fiber concentration increased after the treatment protocol both in the vulvar skin and in the vaginal wall: elastin in the vaginal wall, 11.4%, and in the vulvar skin, 61%; collagen in the vaginal wall, 26%, and in the vulvar skin, 27%. The current study demonstrated the efficacy and safety of this nonsurgical RF procedure combined with a hyaluronic acid (HA) injection into the skin of the labia majora on clinical, histochemical, and aesthetic levels.

## 1. Introduction

Menopause-related hypoestrogenism negatively affects vaginal and urinary health, often leading to genitourinary syndrome of menopause (GSM) [1]. The lack of estrogens causes floppy, droopy vulvar skin, and an atrophic, pale vaginal epithelium with petechiae, dryness, burning, irritation, and sexual symptoms such as discomfort, pain, and reduced sexual function. This disorder, called vulvovaginal atrophy (VVA), commonly causes urine incontinence, dysuria, urgency, and recurrent UTIs [2]. VVA is a chronic illness that worsens if left untreated, unlike menopausal vasomotor symptoms. According to the latest study, VVA affects 36% to 90% of perimenopausal and postmenopausal women [3,4]. The treatment of VVA comes down to long-acting vaginal moisturizers when there are mild and topical estrogens in moderate to severe VVA [5]. Systemic replacement does not always relieve vaginal dryness symptoms and requires topical estrogen [6,7,8].

Pregnancy and childbirth are thought to cause vaginal laxity [VL] [9]. There is a correlation between objective indicators of pelvic floor function in the postpartum period [10] and the loss of physical feeling and diminished sexual satisfaction following vaginal birth. It is now commonly acknowledged that vaginal delivery can cause trauma to the pelvic floor, particularly the levator ani muscle [11,12,13]. In a worldwide survey of urogynecologists, 83% of 553 respondents identified vaginal laxity as underreported by their patients. Most described it as a self-reported uncomfortable condition that compromises sexual function and relationships [14,15,16]. VL is linked to less vaginal feeling and a poorer sexual life [17]. A total of 46% of women with VL met the criteria for sexual distress and 65% met the criteria for female sexual dysfunction [18].

VL can be treated with Kegel exercises and surgery [9,16]. Vaginoplasty and perineoplasty are often performed for this reason, but like any surgical treatment, there are sometimes complications. Scarring or nerve damage can induce dysesthesia, hypercorrection, and dyspareunia [19,20], hence, the need to look for effective mini invasive treatment.

Radiofrequency and laser technologies can also improve laxity and atrophy of the vaginal wall [21,22]. RF devices increase tissue molecular mobility, causing heat [21]. Monopolar, bipolar, and multipolar RF devices operate on the principle of the radiofrequency current flow through tissue, which resists creating a rising temperature up to 43 degrees centigrade. This heat activates heat shock proteins and initiates the inflammatory cascade, causing fibroblasts to make collagen and elastin. RF was studied in a vaginal sheep model using a monopolar Viveve applicator. Three months later, collagen production and fibroblast development had increased. This ovine model lacked elastin [23]. In a pig model, BTL Industries’ Exilis Ultra 360 monopolar intravaginal applicator also improved vaginal tissue. Neocollagenesis and neoelastinogenesis were identified [24].

In our study (non-published) on swine’s vaginal wall, we evaluated the effects of bipolar RF Sectum 360 with a vaginal applicator (Berger and Kraft Medical). Vaginal radiofrequency (RF) exposure was administered twice every 4 weeks and the vaginas were removed after slaughter, four weeks after the last radio frequency exposure. We demonstrated a substantial rise in the concentration of elastin and collagen fibers in the zones exposed to radiofrequency up to 1.3 mm of the thickness of the vaginal wall. There was a 52.8% rise in elastin concentration and a 103.6% rise in collagen concentration in the RF-exposed areas of the vaginal wall. Studies of women with VL and VVA have shown that RF therapy is safe and effective in improving VL and VVA [25,26,27,28,29,30]. However, the optimal technique of RF therapy is still unclear [28].

The labia majora are also subject to changes characteristic to VVA. With age and parity, the labia majora decrease their volume and tone, and change their coloration because vascularization rapidly decreases after menopause. Following the loss of follicular activity, the external genitalia lose subcutaneous fat, and connective tissue relaxes and becomes less elastic; this is associated with pain during sexual intercourse [31]. Moreover, for these reasons, an increasing interest in the aesthetic appearance of female genitalia has also been observed in the field of cosmetic medicine. In the last years, the industry has developed new dermal fillers for facial and body treatments. Hyaluronic acid (HA) filler is the most common due to its good outcomes, ease of administration, and lack of side effects [32]. Hyaluronic acid (HA) can also be safely injected during a simple outpatient procedure to augment the labia majora, providing youthful and natural results [33,34].

Several studies [35,36,37] have evaluated combined HA and RF treatment regimens. The reason for their combined use results from the fact that RF therapy delivers homogeneous heat to the dermis, which remodels collagen and tightens the skin, but it cannot restore lost volume. In a study of RF treatment on soft-tissue fillers in an animal model, England et al. [37] found that several passes of RF treatment directly over filler-injected skin did not cause immediate adverse reactions and did not significantly affect treatment duration. Goldman et al. [38] reported that humans can safely receive laser, RF, and intense pulsed light therapy after HA therapy.

No study has shown how HA and RF acting together can affect the labia majora skin. To address the limits of both modalities and assume a synergistic impact, our patients were treated with a radiofrequency (RF) device before HA filler injections (filler 28 mg/mL PEG-crosslinked) and, subsequently, with three consecutive vulvar and vaginal RF applications every 3–4 days. Zerbinati et al. [34] found that infiltration of the labia majora with HA-based PEG-crosslinked filler is reproducible, complication-free, reversible, and 96.6% patient-satisfying.

We predicted RF therapy can boost HA’s efficacy, especially in the vulvar region. This study evaluated the efficacy and safety of RF therapy combined with HA injection in women with VL and VVA.

## 2. Materials and Methods

Twenty women with VL and VVA aged between 36 and 72 (mean age 53.4) participated in this non-randomized, prospective study. The study was approved by the Research Ethics Committee under number 09/KB/VII/2020, and conducted according to the guidelines recommended by the 2000 Declaration of Helsinki, updated in 2008. Demographic data, number of deliveries, menopause, and pharmacologic treatments were recorded. Hypotrophy of the labia majora was preoperatively staged according to classification by Fasola considering both the adipose tissue and the cutaneous layer [33]. The inclusion criteria were patients with symptoms of VVA and VL as vaginal and vulvar complaints, vulvar skin laxity and hypotrophy perimenopause, menopause or surgical menopause, and cervical cytology test negative for cancer. The exclusion criteria were use of hormone therapy (either systemic or topical) or long-acting moisturizers within the last 6 weeks prior to the initial assessment; patients with active or recurrent genital infection (e.g., genital herpes, candidiasis, bacterial vaginosis); patients with human immunodeficiency virus; recurrent urinary tract infection; pelvic radiation therapy or brachytherapy; reconstructive pelvic surgery and previous vaginal or vulvar treatment with energy-based devices and/or hyaluronic acid fillers. After history taking and physical examination, patients were selected and instructed about the procedure, and, after giving informed consent and authorization for photographic documentation, they answered the following questionnaires: Female Sexual Function Index (FSFI) and Vaginal Laxity Questionnaire (VLQ). The vaginal status was examined with the use of Vaginal Health Index (VHI). The aesthetic appearance of the vulva was evaluated by Global Aesthetic Improvement Scale (GAIS) and the comfort of the protocol was assessed for each procedure separately by visual analogue scale (VAS). The treatment course consisted of injection of HA into the skin of labia majora, 1–1.5 mL per side followed by 4 vaginal and vulvar applications of radio frequency (RF) with an interval of 3–4 days.

### 2.1. Hyaluronic Acid

In our study, we used a well examined area ‚ the monophasic hydropolymer that was created with 28 mg/mL of HA crosslinked with polyethylene glycol (PEG) (Neauvia^®^ Intense Rose, Matex Lab, Switzerland) [34]. PEGylation also seems to offer considerable advantages in the field of fillers for aesthetic use, both in terms of safety and of gel performance [39,40,41]. Both PEG and hyaluronic acid are polymers, and their union allows creation of matrices with scaffold architecture; that is, a three-dimensional weft consisting of large meshes that offer high assimilation of the gel into the tissue, and the possibility to include and gradually release molecules that are useful for skin rejuvenation [41,42,43].

The procedure involves administering HA filler to the subcutaneous tissue of labia majora (between tunica dartos and fibrous layer of an adipose sac) through an injection, using an 18 G cannula. Blood vessels and nerves supplying the vulva are located within the lower pole of labia majora, therefore caution is advised when the first injection is carried out at the lower pole. The depth of injection plays an important role, as accessing too deep structures may result in administering the filler to the adipose sac, thus disrupting its effect.

The first step involves a decontamination and anesthetizing of the area to be injected. Local anesthesia is used with approximately 2 mL of 2% lidocaine administered along the labia in a single injection per side. Firstly, a 16 G needle is used to access the labia. Then, the 18 G cannula is used to administer the filler to the lower pole of the labia. A linear, upward application mode should be followed. It is important to administrate the product in the whole length of the labia in the way that enables creation of a natural shape by dividing this as one-fourth of the syringe at the top pole, two-fourths in the middle, and one-fourth at the lower pole. No more than 1.5 mL of hydrogel 28 mg/mL PEG-crosslinked HA was injected on each side for all the patients treated.

### 2.2. Radiofrequency (RF)

We used a disposable gynecological applicator with a bipolar radio frequency Sectum 360 degrees (Berger and Kraft Medical). The RF therapy schedule included four treatment sessions separated by three to four days. Each therapy session included vaginal and vulvar application (labia majora and perineum).

During the vulvar treatment, the current is released in a continuous mode and the applicator is set in motion mode using slow circular movement over the vulvar skin for 10 min (both sides). The energy set up was between 5–10 W and was adjusted based on patient’s feedback (comfortable or uncomfortable).

The intravaginal application involves placing an applicator to the vaginal canal behind the hymenal ring and warming vaginal wall over the length of approximately 10 cm over the course of 3–7 min. The energy setup was between 15–20 and, similarly to vulvar application, was adjusted based on patient’s feedback. Unlike vulvar application, in the vaginal procedure, the current was released in a pulse mode (500 ms), which means that there was no need to move the applicator back and forth in a vaginal canal, which can be embarrassing for both a patient and a doctor performing a procedure. To affect the vaginal wall evenly, the vagina was divided into three 3 cm sections, and when performing a procedure, the tip of an applicator (two rings part) was placed for 3 min in each section.

Before each RF procedure, a specially designed for this purpose gel, containing glycerin without no water, was applied (Neauvia Gel).

### 2.3. Histochemical Analysis

To measure changes in collagen and elastin concentration, specimens were obtained from one patient under local anesthesia with 2% lidocaine (lignocaine 2% 20 mg/mL Polfa Warszawa S.A.) Punch biopsies of the skin of labia majora and vaginal wall in the RF-exposed areas were used to gather samples before and six weeks after the last RF application. The samples were stained to look for collagen and elastin concentration according to the included staining procedure instructions (Mallory Trichrome (Bio Optica, Milan, Italy, cat. no.: 04-020802), and orcein Bio Optica, Italy, cat. no.: 04-055802, Sirius red picrate, Italy, cat. No.:04-121873) Each specimen was computationally processed by the KS 300 (Zeiss) scanner and evaluated according to the selected region of interest. The evaluated area embraced the vaginal wall layers just under the lamina propria of the vaginal epithelium (up to 1.3 mm of the thickness of the vaginal wall). We used a magnification of 400×. Smaller magnifications would embrace the layers beyond the zone of interest and would interfere with the measurement. After threshold values were established, the recorded pictures were segmented by counting the areas of the objects designated for segmentation. Quantitative measures were performed by the summation of separate areas in pixels. Due to the fact that values of segmented areas in pixels were extremely high, a logarithmic scale was used.

## 3. Results

### 3.1. Outcome Measures and Statistic Evaluation

The Friedman test, anon-parametric equivalent of the repeated measures ANOVA, was used to compare the outcomes (numerical variables) between data points. In post hoc analysis, the Wilcoxon signed rank test was used for pairwise comparisons between all time points. For qualitative data, Cochran’s Q test followed by McNemar’s post hoc procedure were used. False Discovery Rate (FDR) adjustment was used to account for multiple comparisons. FDR was calculated using the Benjamini–Hochberg method. An FDR-adjusted *p* value < 0.05 was considered significant. Statistical analysis was performed and figures created using R statistical environment (R Core Team (2021). R: A language and environment for statistical computing. R Foundation for Statistical Computing, Vienna, Austria. URL https://www.R-project.org/ (accessed on 1 September 2022).

The sample size was calculated using GPower (3.1.9.6). A sample size of n = 20 was sufficient to detect large effects (corresponding to eta-squared of 0.14), assuming alpha error 0.05, power of 80%, number of groups 1, and number of measurements 4.

### 3.2. VL

**Figure 1:**
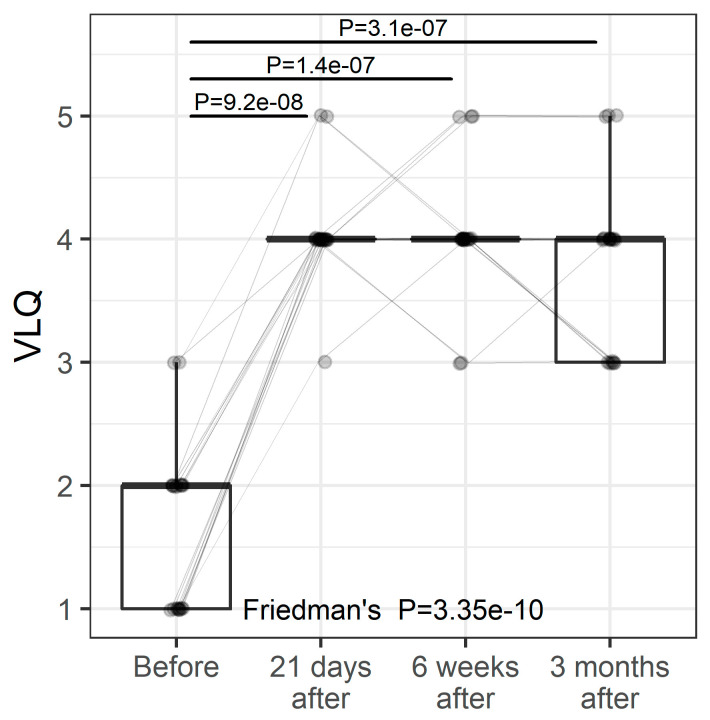
VLQ at study entry and follow-up visits (individual values and summary statistics). Individual values are represented by points joined by lines. Summary statistics are shown using box plots. The middle line of the box plot is median, the lower and upper hinges correspond to the first and third quartiles (the 25th and 75th percentiles). The upper whisker extends from the hinge to the largest value (no further than 1.5*IQR from the hinge, IQR is the interquartile range—distance between the first and third quartiles). The lower whisker extends from the hinge to the smallest value (no further than 1.5*IQR of the hinge). Median line overlaps with lower and upper hinges. *p*-values were determined using Friedman’s test, followed by pairwise post hoc Wilcoxon test with FDR adjustments for multiple testing correction.

Vaginal laxity was assessed by a non-standardized subjective vulvovaginal laxity questionnaire (VLQ) using a 7-point Likert scale. Participants were eligible if they self-reported a perception of vaginal introital laxity defined as ”very loose”, “moderately loose”, or “slightly loose” on a seven-point Likert scale [17,18,27]. Data were collected before the first treatment and during the consecutive follow-up visit. The average improvement was calculated. VL changed significantly over time (Figure 1, *p* < 0.0001). Post hoc comparisons revealed that significant differences were found between study entry and follow-up visits but not between follow-up visits.

### 3.3. FSFI

**Figure 2:**
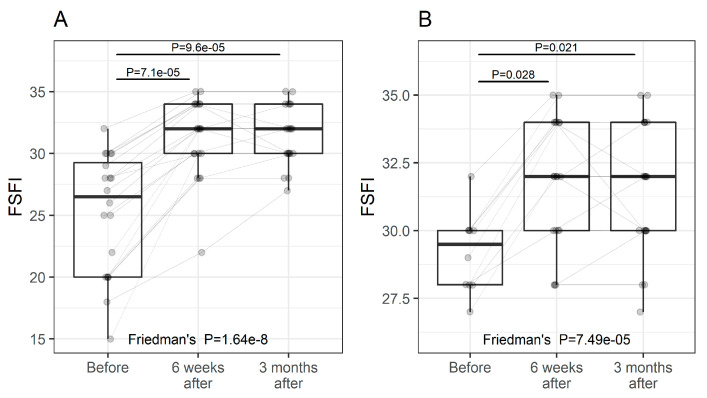
FSFI at study entry and follow-up visits (individual values and summary statistics). (**A**)—all participants, (**B**)—participants with FSFI score > 26. Individual values are represented by points joined by lines. Summary statistics are shown using boxplots. The middle line of the boxplot is median, the lower and upper hinges correspond to the first and third quartiles (the 25th and 75th percentiles). The upper whisker extends from the hinge to the largest value (no further than 1.5*IQR from the hinge, IQR is the interquartile range—distance between the first and third quartiles). The lower whisker extends from the hinge to the smallest value (no further than 1.5*IQR of the hinge). *p*-values were determined using Friedman’s test, followed by pairwise post hoc Wilcoxon test with FDR adjustments for multiple testing correction.

The FSFI is a widely accepted, global evaluation used in female sexual medicine trials [44]. The FSFI is a 19-item questionnaire divided into six domains: desire, arousal, lubrication, orgasm, satisfaction, and pain, which evaluate a woman’s recent state of sexual function (within the past 4 weeks). The domain scores combine to create a total score (range 2–36). A change in the FSFI during the study was significant (Figure 2a, *p* < 0.0001). A six-week follow-up demonstrated a significantly improved FSFI score (from 26.5 (9.3) to 32 (4), median (IQR), *p* < 0.0001) and remained significantly better at the 3-month follow-up (32 (4), *p* < 0.0001 versus baseline, Figure 2). A total FSFI score of 26 is recognized in the medical literature as indicating female sexual dysfunction (FSD). We also observed significant improvements in women without FSD (Figure 2b).

We also defined a clinically significant change in women with no FSD, Figure 2b, for an FSFI posttreatment total score of at least 2 points regardless of the participant’s score at study entry. We based this on a similar calculation and methodology to that previously reported [45,46]. All participants exhibited a clinically significant change with a median change of 5 and 50% of women ranging from 4 to 8.

### 3.4. VHI

**Figure 3:**
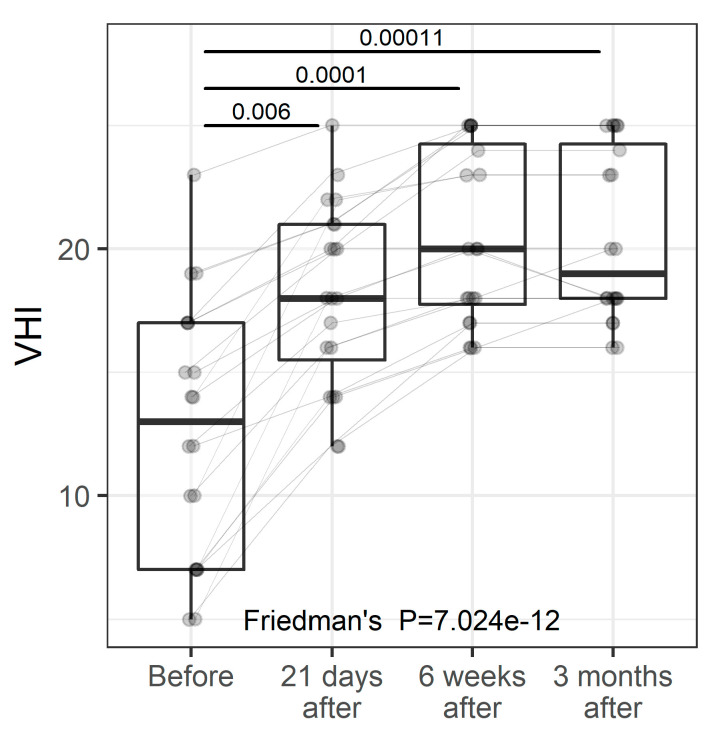
VHI at study entry and follow-up visits (individual values and summary statistics). Individual values are represented by points joined by lines. Summary statistics are shown using boxplots. The middle line of the boxplot is median, the lower and upper hinges correspond to the first and third quartiles (the 25th and 75th percentiles). The upper whisker extends from the hinge to the largest value (no further than 1.5*IQR from the hinge, IQR is the interquartile range—distance between the first and third quartiles). The lower whisker extends from the hinge to the smallest value (no further than 1.5*IQR of the hinge). *p*-values were determined using Friedman’s test, followed by pairwise post hoc Wilcoxon test with FDR adjustments for multiple testing correction.

Vaginal health linked to VVA was evaluated by the Vaginal Health Index (VHI). The VHI consists of the clinical analysis during the specular examination of five parameters and is graded from 1 to 5. The sum of the values of the parameters evaluated results in the total vaginal health score. The VHI gives scores of vaginal moisture, vaginal fluid volume, vaginal elasticity, pH, and vaginal epithelial integrity. The lower the score, the higher the atrophy. The sum of the values of the evaluated parameters results in the total vaginal health score. When the overall score is less than 15, the vaginal mucosa is considered atrophic [47,48]. VHI was measured at baseline and three follow-up visits (Figure 3). Overall, there was a significant improvement in the VHI score over time with the greatest and most significant change taking place between the baseline and 21-day follow-up (*p* = 0.006). The VHI score leveled off after the 21-day follow-up and no significant changes were found between follow-up visits thereafter.

### 3.5. GAIS

**Figure 4:**
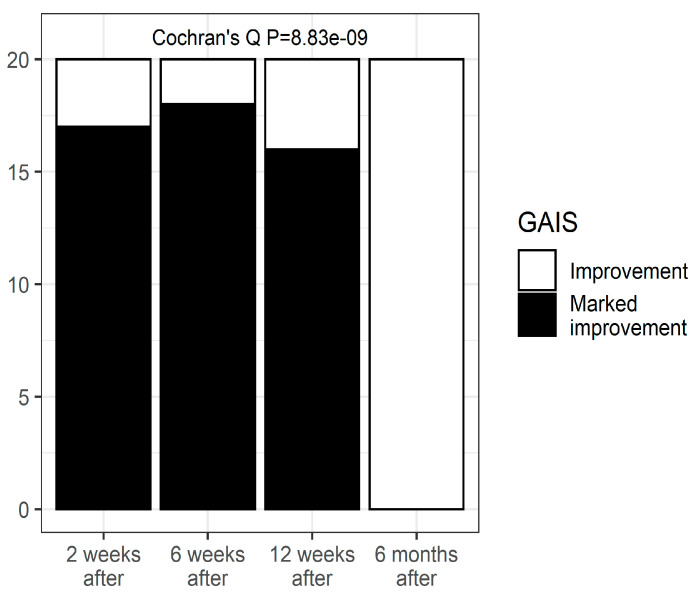
The aesthetic improvement in vulva or lack of it on the Global Aesthetic Improvement Scale (GAIS).

The patient rated the aesthetic improvement in the vulva or lack of it on the Global Aesthetic Improvement Scale (GAIS) ranging from −1 (worsening) to 5 (very much improved) at 6 weeks after the procedure and 12 weeks after the procedure with a preoperative digital picture. Patients were asked to write their score on a form, analogously identified by a number from the GAIS. The forms were collected and analyzed once all evaluations were accurately recorded at the interval follow-up visits at 6 and 12 weeks and 6 months. There were only two categories of the GAIS score: an improvement and a marked improvement. Their distribution changed significantly with time (Figure 4, *p* < 0.0001). Marked improvement was found in 85% at 2 weeks, 90% at 6 weeks, and 80% of participants at 12 weeks, and in none of the participants 6 months after. The post hoc analysis revealed that the percentages of marked improvement were significantly higher when compared with the 6-month follow-up (0.000112, 0.000112 and 0.000127 for 2 weeks, 6 weeks, and 12 weeks, respectively) but not between each other.

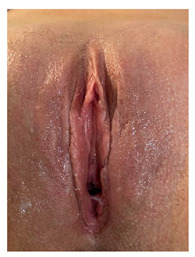

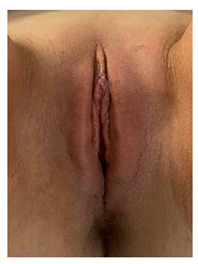
Clinical case 1 before and after the procedure.
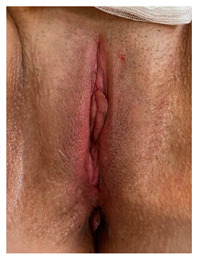

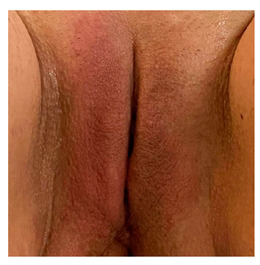
Clinical case 2, before the and after the procedure.
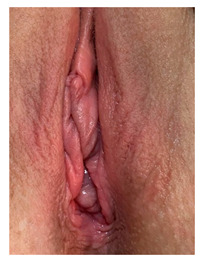

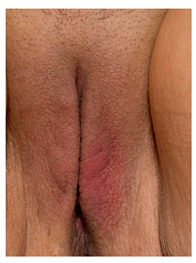
Clinical case 3, before and after the procedure.

### 3.6. VAS

**Figure 5:**
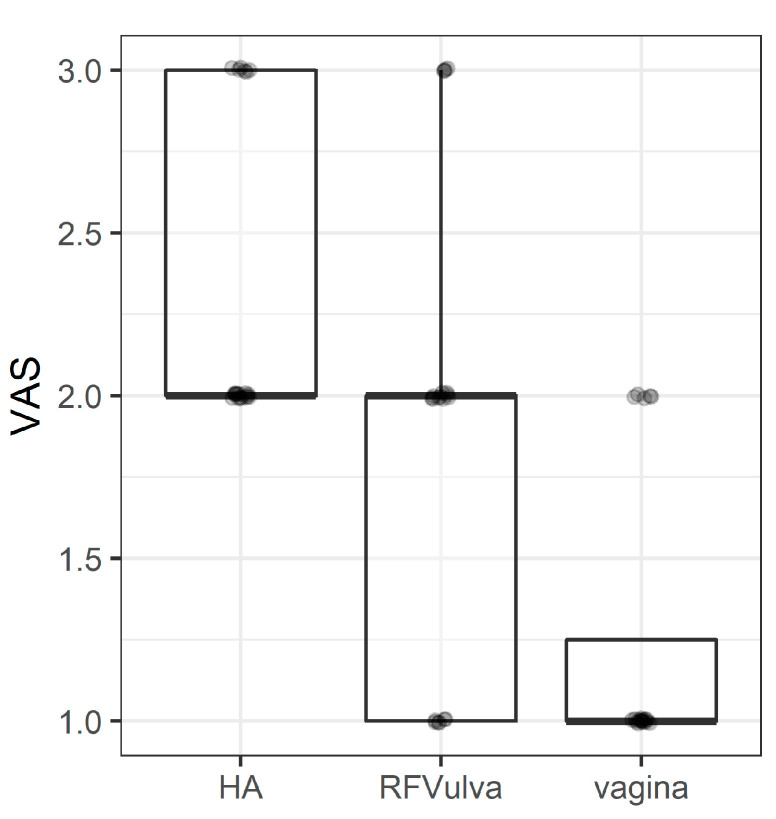
The visual analog scale (VAS) to evaluate analgesic effects of various therapies. The middle line of the boxplot is median, the lower and upper hinges correspond to the first and third quartiles (the 25th and 75th percentiles). The upper whisker extends from the hinge to the largest value (no further than 1.5*IQR from the hinge, IQR is the interquartile range—distance between the first and third quartiles). The lower whisker extends from the hinge to the smallest value (no further than 1.5*IQR of the hinge). Median line overlaps with lower and upper hinges.

A preponderance of evidence demonstrates that the visual analog scale (VAS) is by far the most frequently used assessment instrument to evaluate the analgesic effects of various therapies (Figure 5). From a structural standpoint, we believe that anyone who is cognitively capable of grasping the parameters and following instructions from a doctor can utilize the VAS. Indeed, the VAS’s success is typically credited to it being simple and convenient in a hectic clinical environment [49,50].

### 3.7. Histochemical Staining Results

The paragraph gives an explanation of the charts (Figure 6, Figure 7, Figure 8, Figure 9, Figure 10, Figure 11, Figure 12, Figure 13, Figure 14, Figure 15, Figure 16, Figure 17, Figure 18, Figure 19, Figure 20, Figure 21, Figure 22 and Figure 23). The *y*-axis indicates the intensity of the staining reaction in the histological preparation; the units and scale are assigned routinely and automatically by the imaging analyzer (KS 300) and this applies to the 2D and 3D diagrams for all histochemical methods used. This allows not only a quantitative comparison of the histochemical reaction but also their specific distribution in the tissue structures, and this is visible in the 3D graphs.

## 4. Discussion

Over the past decade, there has been an increase in the use of energy-based devices for sexual dysfunction, vulvovaginal laxity, and GSM, as well as to improve the appearance of external genitalia. Recently, the use of radiofrequency for the treatment of vaginal laxity, sexual dysfunction, and genitourinary syndrome of menopause (GSM), has shown promising treatment outcomes [25,26,29].

As a part of a urogenital function, the appearance deteriorates too. There are two studies showing the efficacy, effectiveness, and safety of an injection of HA into the skin of the labia majora [33,34] as well as the combination of the treatment in the region of the nasolabial folds and perioral rhytides with monopolar radiofrequency [37].

In our study, RF therapy performed four times, combined with HA injection improved all evaluated domains statistically significantly: vaginal laxity, female sexual disorder, sexual function, and genitourinary symptoms of menopause and labia majora hypotrophy. We also showed at the histochemical level an increase in collagen and elastin concentrations in both the vaginal wall and vulvar skin. The efficacy, safety, and comfort of the procedure were similar to other studies quoted above [25,26,27,29].

What distinguishes our research is the use of a bipolar radiofrequency and combining it with an HA injection into the skin of the labia majora to enhance the aesthetic effect. We have not encounter this combination of RF and HA in the skin of the labia majora in the literature.

In our opinion using bipolar RF seems to be safer then monopolar (the current goes between two rings, not between the active electrode and grounding pad under a patient’s back , and as a result, a current flows in a not too well defined area). Another facet is the possibility of launching an RF current out of the handpiece in two ways: a pulse mode and a continuous mode. When we use external, vulvar application, the continuous mode, in motion, is recommended and we used it in our protocol.

The pulse mode is devised to be used in a vaginal application with no need to move the handpiece. With the monopolar devices accessible on the market, there is a need to move the vaginal handpiece back and forth in the vaginal canal, which can be embarrassing for the patient and the doctor performing the procedure.

The combined treatment turned out to be safe and painless with no side effects and with a statistically significant improvement between the baseline and findings 3 months after treatment. Apart from clinical efficacy, we also exhibited an aesthetic effect, evident in the pictures before and after, as well as evaluated by the GAIS.

## 5. Conclusions

The current study demonstrated the efficacy of this nonsurgical RF procedure combined with a hyaluronic acid (HA) injection into the skin of the labia majora on clinical, histological, and aesthetic levels.

Our study provides further evidence for the clinical utility, and direction for further evaluation. Since this is a pilot study with a small number of patients, further studies are required to corroborate our findings and evaluate the long-term effects of the combined treatment of hyaluronic acid and radiofrequency.

## Figures and Tables

**Figure 6:**
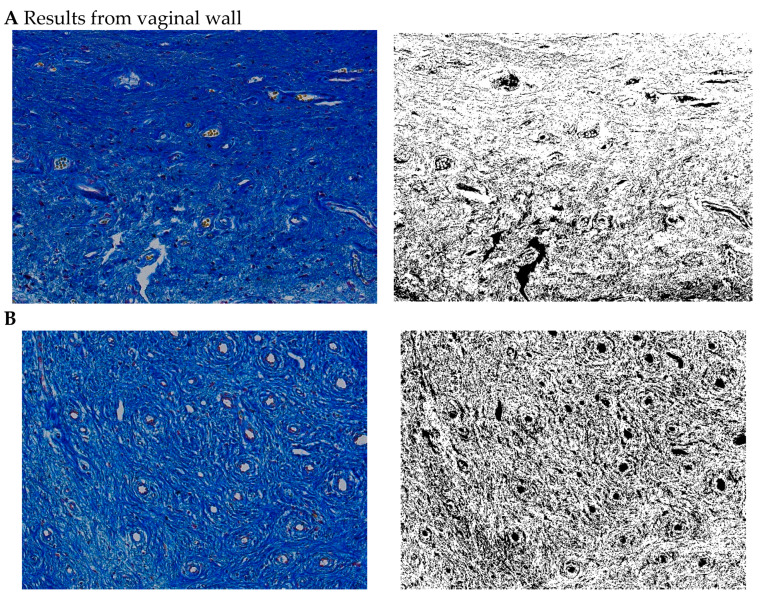
Segmentation before (**A**) and after (**B**) treatment (Mallory trichrome).

**Figure 7:**
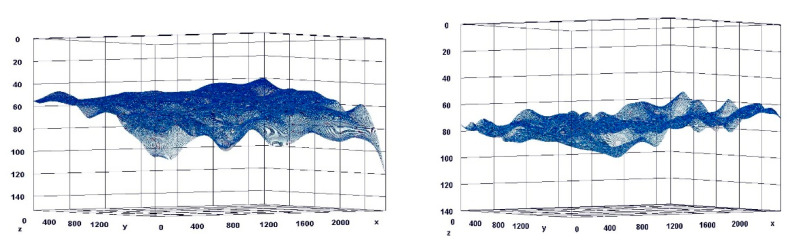
Visualization of the differences in collagen concentration before and after treatment (computer program IMAGEL).

**Figure 8:**
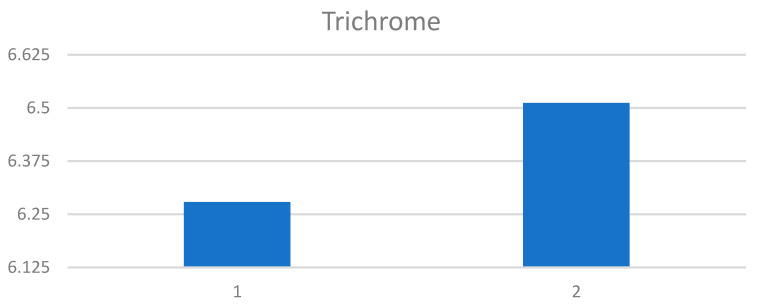
The comparison of specimens before and after the treatment revealed 26.6% rise in collagen concentration.

**Figure 9:**
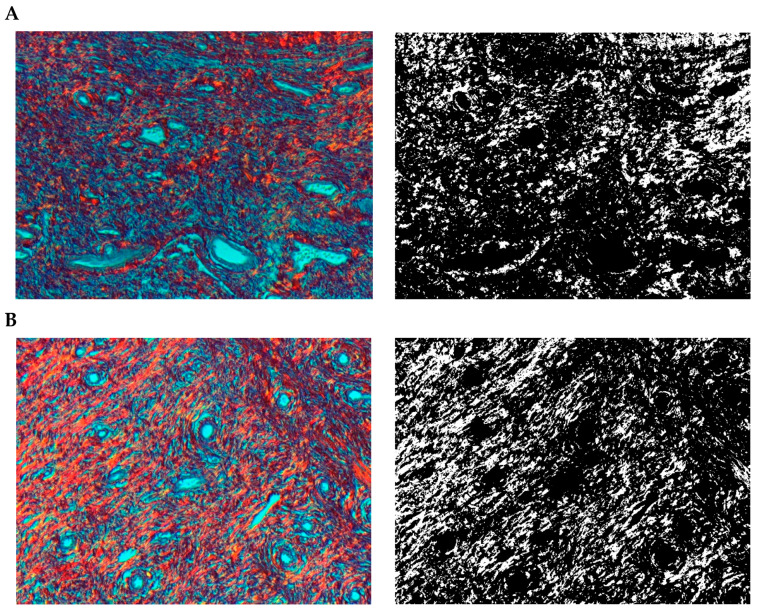
Segmentation before (**A**) and after (**B**) treatment (picrosirius red staining).

**Figure 10:**
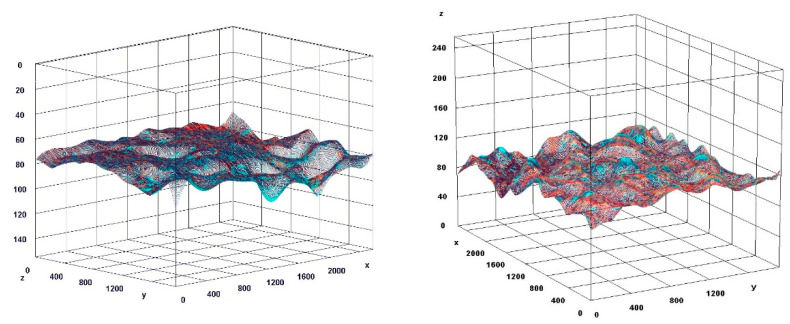
Visualization of the differences in collagen concentration before and after treatment.

**Figure 11:**
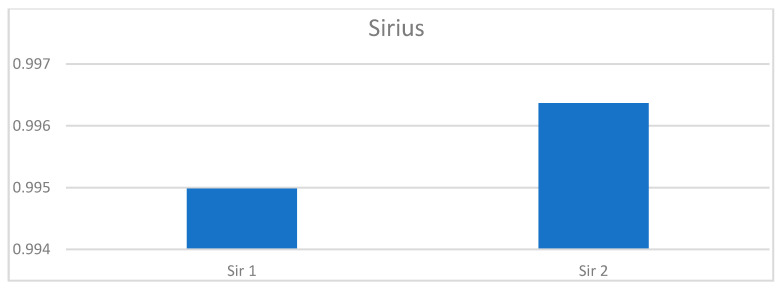
The comparison of specimens before and after the treatment revealed 26.4% rise in collagen concentration.

**Figure 12:**
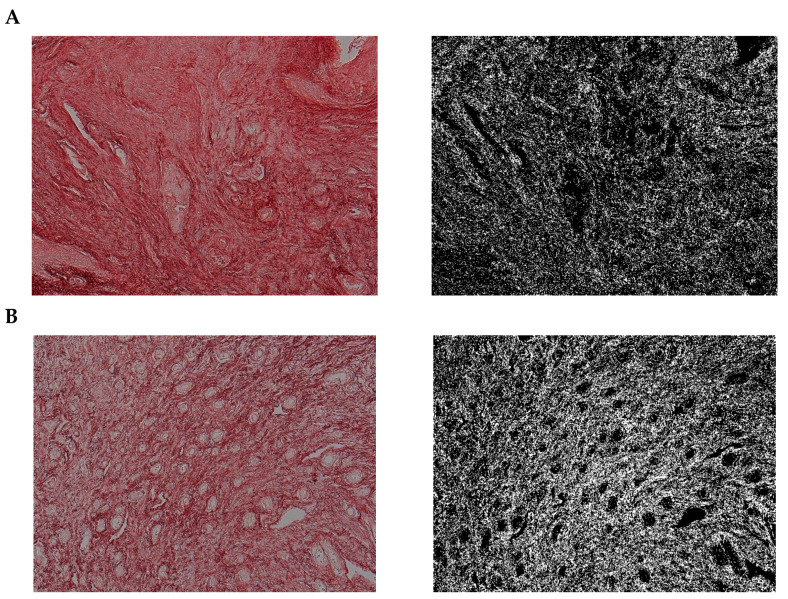
Segmentation before (**A**) and after (**B**) treatment (orcein).

**Figure 13:**
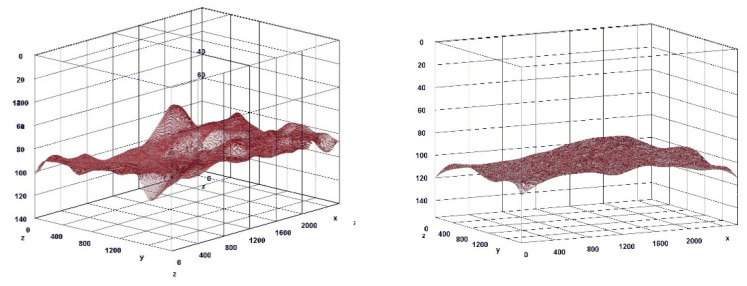
Visualization of the differences in elastin concentration before and after treatment.

**Figure 14:**
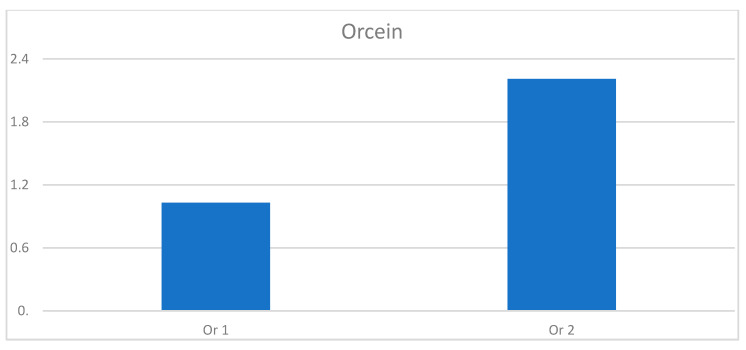
The comparison of specimens stained with orcein before and after the treatment revealed 11.43% rise in elastin concentration.

**Figure 15:**
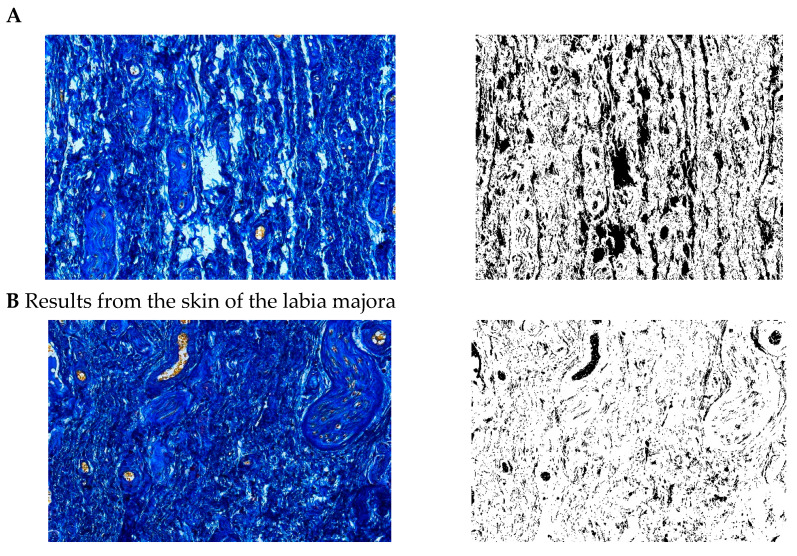
Segmentation before (**A**) and after (**B**) treatment (Mallory trichrome).

**Figure 16:**
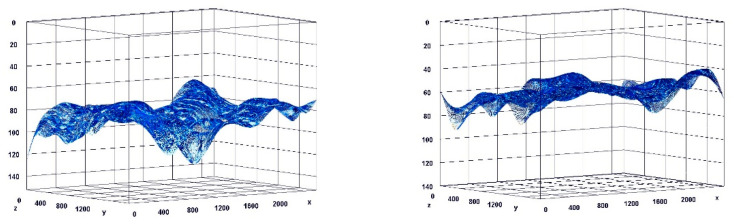
Visualization of the differences in collagen concentration before and after treatment (Trichrome).

**Figure 17:**
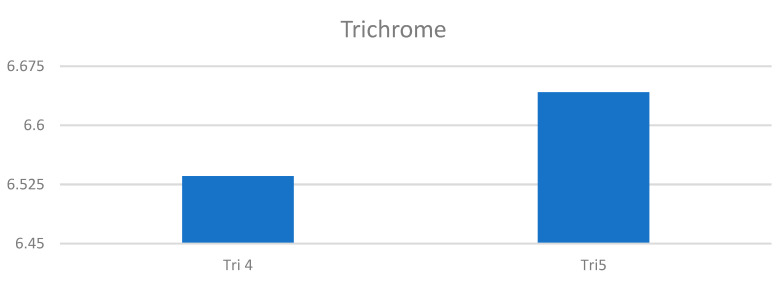
The comparison of specimens stained with trichrome before and after the treatment revealed 27.8% rise in collagen concentration.

**Figure 18:**
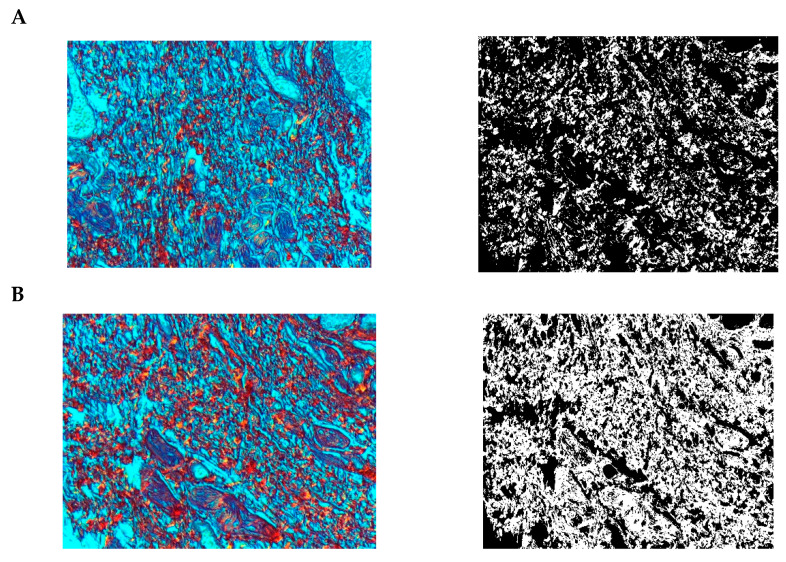
Segmentation before (**A**) and after (**B**) treatment (picrosirius red staining).

**Figure 19:**
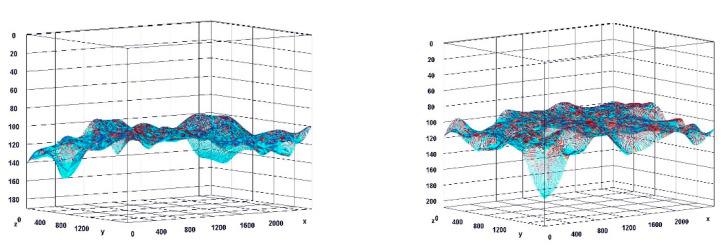
Visualization of the differences in collagen concentration before and after treatment.

**Figure 20:**
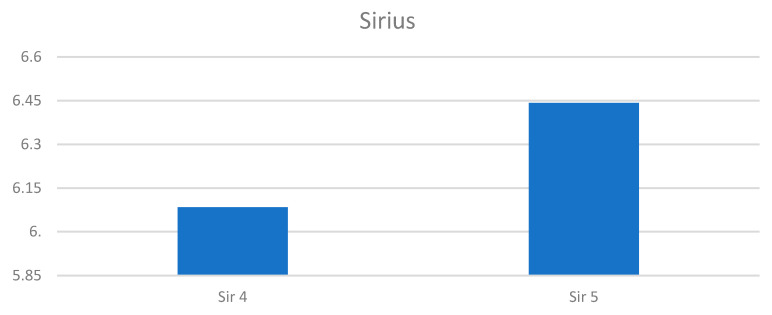
The comparison of specimens stained with trichrome before and after the treatment revealed 26.7% rise in collagen concentration.

**Figure 21:**
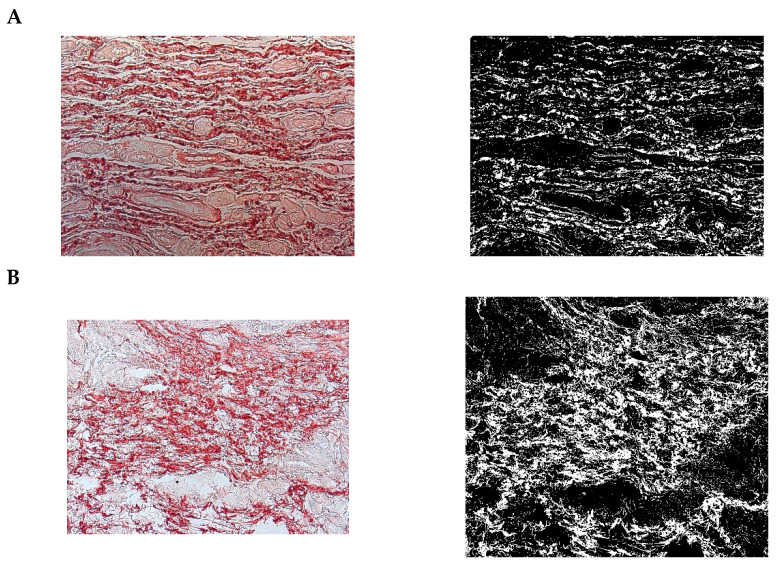
Segmentation before (**A**) and after (**B**) treatment (orcein).

**Figure 22:**
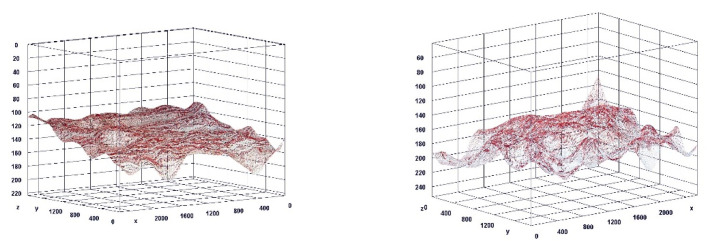
Visualization of the differences in elastin concentration before and after treatment.

**Figure 23:**
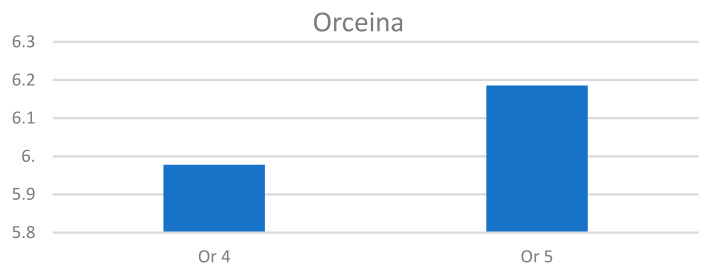
The comparison of specimens stained with orcein before and after the treatment revealed 61.8% rise in elastin concentration.

## Data Availability

Not applicable.
